# Research on the Coupling Coordination Relationships between Urban Function Mixing Degree and Urbanization Development Level Based on Information Entropy

**DOI:** 10.3390/ijerph18010242

**Published:** 2020-12-31

**Authors:** Xuanxuan Xia, Kexin Lin, Yang Ding, Xianlei Dong, Huijun Sun, Beibei Hu

**Affiliations:** 1 School of Business, Shandong Normal University, Jinan 250358, China; 20113017@bjtu.edu.cn (X.X.); 201724040211@stu.sdnu.edu.cn (K.L.); 2017020961@stu.sdnu.edu.cn (Y.D.); dongxianlei@sdnu.edu.cn (X.D.); 2 School of Economics and Management, Beijing Jiaotong University, Beijing 100044, China; 3 Key Laboratory of Transport Industry of Big Data Application Technologies for Comprehensive Transport, Ministry of Transport, Beijing Jiaotong University, Beijing 100044, China; hjsun1@bjtu.edu.cn

**Keywords:** POIs’ spatial entropy, taxis’ temporal entropy, urbanization development level, coupling coordination degree

## Abstract

With the rapid development of urbanization, the blind expansion of urban space has led to a series of social problems. In this process, the degree of urban function mixing affects the urbanization development level, making it particularly important to study the degree of coupling coordination between the two aspects. In this paper, taking Beijing as an example, we use urban point of interest (POI) data and taxi GPS trajectory data to calculate the urban POIs’ spatial entropy and taxis’ temporal entropy, based on the information entropy. We use the POIs’ spatial entropy and taxis’ temporal entropy to measure the urban function mixing degree. Also, the model of coupling coordination degree is used to measure the degree of coupling coordination between the urban function mixing degree and the urbanization development level. The results indicate the following: First, the POIs’ spatial entropy and taxis’ temporal entropy have significant regional imbalances. On the whole, both show a declining pattern when moving from the central urban area to the outer suburbs. The urban function mixing degree and urbanization development level are also higher in the central urban area than in the outer suburbs. Second, the coupling coordination among the urbanization development level, POIs’ spatial entropy, and taxis’ temporal entropy is distributed unevenly across various regions, which means that the three types of coupling coordination are in balanced development in the central urban area, but in unbalanced development in the outer suburbs. Third, from the perspective of spatial correlation characteristics, the higher is the degree of spatial agglomeration, the higher are the urban function mixing degree and urbanization development level, and the higher is the coupling coordination degree among the urbanization development level, POIs’ spatial entropy, and taxis’ temporal entropy. Therefore, relevant departments should plan the construction of urban functional areas reasonably, according to the degree of coupling coordination between the urban function mixing degree and the urbanization development level in different regions, so as to realize the healthy and sustainable development of a city.

## 1. Introduction

The environmental and economic problems caused by the continuous expansion of urban land have become one of the most difficult challenges in the sustainable development of cities all over the world. After World War II, the expansion of buildings in many countries accelerated and settlements became more scattered. For example, according to the European Environment Agency’s report entitled “European Urban Sprawl” in November 2016, Belgium is one of the EU countries most affected by urban sprawl [1]. Coincidentally, metropolitan areas began to appear in the United States in the 1860s, connected by a highway that crossed ten states. The fact that the growth rate of urban areas is faster than the population leads to a decline in urban density. Consequently, some city issues like urban imbalance problem occur. Specifically, the extensive and inefficient urban construction has caused significant waste of land resources. At the same time, the construction of functional areas and the spatial structures in different areas of cities are lagging behind, in a way that is clearly inconsistent with the level of urbanization. Generally, both the construction of functional areas in central urban areas and various social infrastructures are relatively complete, while the construction of functional areas in the outer suburbs is lagging behind, relatively.

In the process of rapid urbanization in China, there has been regional incongruity between the urbanization level and the spatial structure of urban functional areas. By the end of 2019, China’s urbanization level had reached 60%. However, the blind expansion of urban space has led to a series of problems, such as low overall urban land resource utilization, unreasonable spatial structure layout, and the waste of resources [2]. “Land urbanization” (land urbanization is due to the advancement of urbanization, the transformation of land use from agricultural to urban construction, and the process of moving land property rights from rural collective to state-owned) has appeared in many cities in China. In response to the inconsistency between the urbanization development level in China and the spatial layout of urban functional areas, the State Council has issued a “National New Urbanization Plan (2014–2020)”, which requires continuous optimization of the urban internal spatial structure layout and improvement of land use efficiency. The development of “compact cities” emphasizes coordination between high-density mixed land use construction and the urbanization level [2].”Compact city” refers to a high-density and diversified city, achieved through the mutual superposition of urban functions so as to increase resource utilization efficiency and promote the sustainable development of the city [3]. Based on the value orientation of the “compact city” [4], China should continue to improve the mixed state of urban functional areas and the land utilization rate. In this way, a shift from city expansion to quality improvement will be realized, and coordinated development of urban functional areas and the urbanization level will be promoted.

China has carried out precise urbanization management for many years, but has still not fundamentally solved the problem of the uncoordinated development between the construction of urban functional areas and the urbanization level. By the end of 2018, according to the Statistics Bureau, the urbanization levels of Shanghai and Beijing had reached 88.1% and 86.5%, respectively, but both have experienced a serious lack of coordination in urban function and use of space. For example, in both cities the fastest development and most concentrated urban construction always occurs in the central urban area, and the highest-end service industry is also concentrated here. In contrast, the development of the suburban counties is relatively backward. Thus, the overall development of these cities is imbalanced, and the mismatch of urban functions and space is serious. One of the most critical reasons for this situation is that it is impossible to accurately measure and quantify the degree of inconsistency in the distribution of urbanization development and urban functional areas in different regions. Because cities are huge and complex, there are obviously regional differences within them, and the spatial function composition and degree of mixing are also difficult to fully measure and quantify. This leads to an inability to fully understand the factors that hinder urbanization development and also affect the rationality of urban planning and layout.

Therefore, we carry out the following two analyses: First, we use urban point of interest (POI) data and taxis’ GPS trajectory data to measure the urban function mixing degree, and quantitatively analyze the construction of urban functional areas in Beijing. Second, we take different areas of the city as the research objects, study the coordinated development relationship between the urbanization development level and the urban function mixing degree, and analyze the influencing factors, so as to provide theoretical support for urban planning and construction. The study indicates that most of the urban central areas are in balanced development, while the outer suburbs are in unbalanced development. According to the results, government departments can strengthen the land use of the central areas of cities and improve the utilization efficiency of functional areas, while also improving the infrastructure and land resource utilization of the outer suburbs and the construction of various functional areas, so as to eventually achieve a reasonable urban space layout and meet the goal of optimizing urban management. It is of great significance to be able to accurately measure and quantify the degree of inconsistency between the urbanization development level and distribution of urban functional areas in different regions. On the one hand, this calculation and the analysis results have important practical significance for urban residents’ transportation, land use improvement, and urban planning and layout. On the other hand, they can provide important data support for the travel efficiency analysis of urban traffic conditions, land use evaluation calculations, and urban layout optimization analysis.

The rest of this paper is composed as follows: Section 2 reviews the literature on the urban function mixing degree and the urbanization development level. Section 3 describes the urban POI data, taxis’ GPS trajectory data and the selection of an evaluation index for the urbanization development level. Section 4 puts forward the method for measuring the urban function mixing degree, the coupling coordination degree model, and the spatial autocorrelation model. Section 5 describes, analyzes and discusses the experimental results. Section 6 summarizes the research described in this article and prospective future work.

## 2. Literature Review

In recent years, with the development of computer technology and the widespread application of mobile smart terminals, people have begun to pay attention to relevant research on the urban function mixing degree and urbanization development level. On the one hand, based on multi-source data such as mobile phone signaling [5], POI distribution, taxis’ GPS trajectories [6,7,8,9,10] and so on, scholars can study the urban function mixing degree, that is, the degree of perfection in the construction of urban multifunctional areas. This can provide theoretical support and a basis for quantitative analysis of the urban land utilization rate, better analysis of and solutions to urban complex problems, and the improvement of urban comprehensive capabilities. At the same time, people are beginning to pay attention to the coordinated relationship between the urban function mixing degree and urbanization development. Studies find that the two are closely related, prompting guidelines and suggestions for the rational planning of urban layouts to improve land use efficiency and urban comprehensive capabilities.

### 2.1. Calculation and Analysis of the Urban Function Mixing Degree

In measuring the urban function mixing degree, scholars mainly calculate and analyze it from two aspects. Firstly, in terms of data, scholars use multiple heterogeneous sources to identify and evaluate the construction of urban functional areas. For example, Gao, Q and Wu, Q used vehicle trajectory data to classify urban functional areas [11,12], and combined this with the density of POIs to determine the spatial distribution and combination of urban functional areas [13]. Long Ying et al. [14] used Sina Weibo’s POI data and check-in data to quantitatively evaluate the function mixing degree, and measured the function mixing degree in a specific area.

Secondly, in terms of research methods, scholars use different measurement methods for the urban function mixing degree, mostly objective weighting methods, such as the entropy method, spatial entropy, mean square deviation, or land use mixing index method. These objective evaluation methods avoid the subjective judgments of researchers. For example, Zhao used the entropy weight and mean square deviation method to measure the functional intensity of urban development land [15], and studied the urban function mixing degree in different areas [16]. Ying et al. calculated the urban function mixing degree in each grid with the land use mixing index proposed by Frank, thereby quantitatively evaluating the function mixing degree [17]. With a modified Bayes theorem and spatial analysis and spatial statistics, Liu built a probabilistic model to infer buildings’ mixed functions, in order to measure the urban function mixing degree [18]. Decraene used spatial entropy to measure the degree of diffusion in functional areas such as residential, commercial and industrial sectors, and quantified the mixing degree of different functional areas in the space with the help of the difference index [19].

Scholars have obtained different results and made different discoveries due to using different calculation methods and different structural data. For example, Liu found that the higher the functional mixing degree was, the shorter the distance between the taxi pick-up and drop-off points was [18]. Decraene found that the industrial areas in most cities were highly concentrated and separated from residential or commercial areas [19]. In addition, some scholars use subjective and objective combination weighting methods to measure the urban function mixing degree. For example, taking New York, Barcelona and Bogota as examples, Dovey reviewed the existing methods for measuring functional combinations and rethought the measurement method for the life/work/access triangle, thereby revealing functional combination changes at different scales by means of map technology, and explaining and measuring the functional mixing degrees in different cities [20].

In the previous studies on the measurement of the urban function mixing degree, scholars have mainly focused on the combined state of the urban spatial structure, and have hardly measured the urban function mixing state in terms of the time dimension. The comparison of specific synchronous research is shown in Table 1. Since the city is a complex system, it needs to be fully excavated and analyzed from the perspective of multi-source heterogeneous data. Therefore, using multi-source data, we have made further improvements, based on the POI data of the physical space and taxis’ GPS data in different time periods, to confirm and portray the urban function mixing degree. Thus, we not only measure the objective existing functional areas of the city, but also explore the urban function mixing degree from the perspective of people’s use of different functional facilities. At the same time, previous studies have mainly focused on the identification and evaluation of the overall functional area of the city, but we refine the division of urban areas and conduct in-depth quantitative analysis of the functional mixing degree of a unit area, thereby more accurately evaluating the urban function mixing degree.

### 2.2. Coordination Relationships between Urban Function Mixing Degree and Urbanization Development

Regarding the coordination relationship between the urban function mixing degree and the urbanization development level, scholars mainly conduct research and analysis in terms of the following two aspects: First, scholars have studied the correlation between the urban function mixing degree and the urbanization development level. For example, He found that in areas with a high population density and urban function mixing degree, office-type functional areas should be added [21]. Liu [22] used mobile phone data collected over a week, combined with the land use characteristics of POIs, and integrated this with the spatial-temporal feature clustering method to calculate the density and spatial entropy of POIs, thereby measuring the urban function mixing degree and finding a strong correlation between it and urban development.

Second, scholars have studied the impact of land utilization on the urbanization development level. The degree of agglomeration of population, industry, and land [23] also affects the development of urbanization. Therefore, Zhang proposed comprehensive evaluation indicators based on these three aspects, and used spatial data analysis methods to explore the impact of spatial patterns on the development of urbanization [24]. The results show that the development of urbanization is closely related to the rational allocation of population, industry and land resources, and that it is important to strengthen land use efficiency and increase the urban function mixing degree [24]. Xu studied the coupling coordination relations among population, land, and economic urbanization, and between each pair of them. The research showed that economic urbanization was the main factor affecting the spatial-temporal difference in the overall coupling coordination degree [25]. Lin discussed the spatial distribution of cities in the process of rapid land urbanization and found that the land urbanization in China showed obvious spatial differences. The specific manifestation was that the larger was the city, the faster the land urbanization [26]. Wang constructed an evaluation index system for population urbanization and land urbanization quality, using a coupling coordination model, and the results showed that low-intensity use of land resources was still a key factor restricting the quality of urbanization in Gansu Province [27].

Previous studies on the coordination relationship between urban function mixing degree and urbanization development level are limited to the correlation between the two, as well as the discussion of related factors affecting urbanization development. Also, scholars have all started from the perspective of the city as a whole, while there is almost no research quantifying and measuring the coordinated development relationship between the two from the perspective of different areas of a city. Differences in the economy, infrastructure, industry and policies in different areas of a city have led to differences in the urban spatial structure. Therefore, compact development strategies and urbanization development policies in different areas of a city need to be adapted to local conditions. Therefore, based on the coupling coordination degree model, our research analyzes the coordinated relationship between the urbanization development level and the urban function mixing degree, for different areas within a city. This enriches the theoretical basis between the urbanization development level and the urban function mixing degree to a certain extent and allows us to quantify and spatially analyze the coordination relationship between them.

To sum up, the previous literature has focused on the methods of measuring the urban function mixing degree and various factors affecting the urbanization development level. Although previous studies have achieved good research results, there is little literature on the coordination relationship between the urban function mixing degree and the urbanization development level. The research method of this paper is different from that in the previous literature. First, based on the information entropy model, the POIs’ spatial entropy model and taxis’ temporal entropy model are constructed, and then the POIs’ spatial entropy and taxis’ temporal entropy of each region are calculated to measure the urban function mixing degree. Second, based on the model of coupling coordination degree, we obtain the spatial and temporal distribution of the coupling coordination relationship among the urbanization development level, the POIs’ spatial entropy and the taxis’ temporal entropy. Finally, based on the spatial autocorrelation model, the spatial correlation characteristics of the coupling coordination degree are analyzed. According to the research results, relevant departments can make corresponding decisions on urban planning and rationally plan urban spatial layout, and the results can also provide theoretical support for urban optimization management and sustainable development.

## 3. Data

As China’s political, economic, technological and transportation center, Beijing attracts the attention of the world. In addition, Beijing is a metropolitan area with both a central city (urban area) and vast suburbs and rural areas. The urban functional areas are diverse and complete. Since the reform and development of China, Beijing’s urbanization has developed rapidly, the scale of the city has continued to expand, and its urban functions have been expanded and improved. However, the urbanization development of Beijing is very unbalanced, mainly manifesting in relatively lagged development of suburban areas, a single economic structure and incomplete functions [28]. In the process of its modern urban development, Beijing, being representative of a typical city in China, had reached an 86.5% urbanization level by the end of 2018. However, Beijing’s central urban area has and is still developing rapidly and the outer suburban area slowly. The problems between its urbanization development level and the construction of urban functional areas are exemplary of many cities in China. Therefore, we choose Beijing as an example to study the problem of uncoordinated development between the urbanization development level and the urban function mixing degree in different urban regions, as well as influencing factors, so as to propose corresponding solutions.

There are two main types of research data: (1) Beijing’s taxi GPS trajectory data and Beijing’s POI data, used to calculate the taxis’ temporal entropy (hereafter TTE) and POIs’ spatial entropy (hereafter PSE) and thereby further measure the urban function mixing degree (hereafter UFMD). (2) Evaluation index data for the urbanization development level (hereafter UDL), used to calculate the UDL in various regions in Beijing. Based on these two types of data, this paper studies the coupling coordination relationship between UDL, PSE and TTE, and provides theoretical support for coordinated and sustainable development of cities.

### 3.1. Urban POI Data

POI data mainly consists of descriptions of geographic objects or event addresses, containing basic information about each functional area of a city, and providing a basic description of a city’s structure. With the Application Programming Interfaces (APIs) provided by the Gaode Platform, we use data-mining technology to obtain Beijing POI open source data, with a total of 1,115,891 data points. The POI data have five fields, including the POI name, longitude, latitude, functional zone and administrative district.

The Gaode POI classification includes 23 categories, the specific content of which can be found at the website https://lbs.amap.com/api/webservice/download. According to the keywords in the POI name and classification indicators of Gaode POI, the Beijing POI open source data (urban POI data matched to detailed addresses) are specifically classified into 19 POI types and 16 administrative regions. The types of POI are catering services, road ancillary facilities, addresses of places, scenic spots, companies, shopping malls, transportation facilities, financial insurance, education, motorcycle services, automobile services, vehicle maintenance, car sales, business residences, sports and entertainment, transit facilities, health care, government, and accommodation services. The administrative districts are Dongcheng, Xicheng, Chaoyang, Haidian, Fengtai, Shijingshan, Changping, Shunyi, Tongzhou, Fangshan, Daxing, Miyun, Yanqing, Pinggu, Mentougou, and Huairou.

### 3.2. Taxi GPS Trajectory Data and Preprocessing

The research data used in this paper are the taxi trajectory data from 565,582 orders in Beijing occurring between 22nd and 24th December 2016. Each order consists of a series of trajectory data sets, that is, the longitude coordinates, latitude coordinates, and instantaneous speed of the taxi at the corresponding timestamp, obtained every 6 s. The most relevant data fields are the order ID, the time stamp corresponding to the tracking point, the longitude coordinates, the latitude coordinates, and the instantaneous speed. After obtaining it, we preprocess the taxis’ GPS trajectory data. The processing steps are as follows:

Step 1 Data cleaning. This process mainly includes deleting abnormal data, duplicate data, unreasonable data, and data from outside the study area. The original data included abnormal data such as abnormal records other than 0 and 1 in the taxi’s operational status, and unreasonable data which did not conform to common sense, such as taxi operating speeds faster than 1000 km/h. The study area is Beijing, with a geographical location of 39°26′ N to 41°03′ N and 115°25′ E to 117°30′ E. Therefore, we delete any data outside the study area, such as that in Tianjin (116.8431° E, 39.6268° N) and Hebei (116.2913° E, 39.4558° N). According to the load status of the taxis, we extract their pick-up point and pick-up time, and obtain 506,940 valid orders.
Step 2 Matching addresses and categorizing POIs. First, the latitudes and longitudes of the taxi pick-up points and the coordinate system of the Beijing electronic map are unified into the WGS-1984 geodetic coordinates to ensure consistency. Then, we use the Gaode LBS open platform to perform reverse address resolution on the latitude and longitude of each taxi pick-up point to obtain the detailed address in each case. Since administrative districts and POIs are not only the basic units of urban areas, but also an important part of urban planning and management, we classify pick-up points of the taxis by POI type and administrative district, based on the detailed addresses obtained through the geographic matching and the classification criteria for POIs described in Section 3.1.
Step 3 Dividing up the longitude and latitude grids. In order to better reflect the spatial change law of the UFMD, we divide the study area (Beijing) into longitude and latitude grid cells. In order to determine the size of the grid cells, we referred to previous studies of urban grid division using taxi GPS data. For example, the grid cell in Twin Cities is 2.14 km^2^ [29], that in Wuhan is 0.4 × 0.4 km [30], and those in Harbin, Shanghai, and Montreal are 1 km^2^ [31,32,33]. Considering the density of Beijing’s road network, Beijing is divided into 31,150 grid cells, of size 1 × 1 km [34].
Step 4 Counting the number of taxi orders in each grid cell and administrative district. In step 2, each taxi order is assigned to a corresponding administrative district, and we now count up the number of taxi orders in each administrative district separately. Then, according to the longitude and latitude grid constructed in step 3, the taxi orders from each grid cell are counted up as well.

### 3.3. Selection of Evaluation Index for Urbanization Development Level

Based on research results related to UDL in China [24,25,35], this paper measures UDL based on six criteria layers, which are population urbanization, economic urbanization, social urbanization, spatial urbanization, infrastructure urbanization, and ecological urbanization. According to the systemic, scientific, comprehensive and operable principles of the construction of the index system, a total of 24 index layers are selected, as shown in Table 2. The data come from the “2017 Beijing Statistical Yearbook” and the “Beijing Regional Statistical Yearbook”.

## 4. Methodology

### 4.1. Measuring Spatial Entropy Based on POI Data

Information entropy is a physical concept, introduced by Shannon to measure the disorder of a system, and to measure the complexity and balance between systems [36,37,38]. In the process of urbanization, the POI is one of the core elements of the urban structure and form. We combine the basic principles of information entropy to calculate the PSE based on the Beijing POI data obtained. The specific measurement steps are as follows:

Step 1: Identify the type of POI and count the total number of different POI types. According to the POI classification indicators provided by Gaode Map, urban POIs are divided into 19 categories. The total number of POIs of type *k* in a city is *A_k_* (*k* = 1,2,L 19), and the total number of POIs in the city is *A* $\;\left(A=\overset{19}{\underset{k=1}{\sum }}A_{k}\right)$.

Step 2: Calculate the PSE in the city. This includes two parts: measuring the PSE of each latitude/longitude grid cell and measuring the PSE of each region.

In this paper, the latitude/longitude grid cells are used as the research units, within each of which the total number of POI types does not change. Let $A_{k}^{m}\left(m=1,2,\;L\;M\right)$ be the total number of POIs of type *k* in grid cell *m*, where *M* is the total number of grid cells in Beijing. Then, the proportion $P_{k}^{m}$ of POI type *k* in the grid cell is shown in Equation (1):

(1) $$P_{k}^{m}=A_{k}^{m}/\sum_{k=1}^{19}A_{k}^{m}$$

Based on information entropy, the calculation method for the spatial entropy $H_{S}^{m}$ of grid cell *m* is shown in Equation (2):

(2) $$H_{S}^{m}=-\sum_{k=1}^{19}P_{k}^{m}\times \log P_{k}^{m}$$

Next, the spatial entropy of each region is calculated, according to the region in which each grid is located. The calculation formula for the spatial entropy $H_{i}^{S}\left(i=1,2,\ldots ,16\right)$ in region *i* is shown in Equation (3):

(3) $$H_{i}^{S}=\frac{\sum_{m=1}^{M_{i}}H_{S}^{m}}{M_{i}}$$

where *M_i_* is the total number of grid cells in region *i*.

### 4.2. Measuring Temporal Entropy Based on Taxi GPS Trajectory Data

The more POIs there are in an area, the more demand passengers will have for taxis at different times. Therefore, in a certain period of time, the number of taxi passengers in a certain area can give an indication of the UFMD to a certain extent. Therefore, based on the taxi GPS trajectory data, and according to Equations (1) and (2), we calculate the TTE. The steps are as follows:

First, let $A_{t}^{m}\left(1,2,\ldots ,M\right)$ be the total number of orders in grid cell *m* and period *t*(*t =* 0,1,L 23). Then, $P_{t}^{m}$, orders in grid cell *m* in period *t* as a proportion of the orders in grid cell *m* across all periods, is as shown in Equation (4):

(4) $$P_{t}^{m}=A_{t}^{m}/\sum_{t=0}^{23}A_{t}^{m}$$

Based on the information entropy, the calculation of the TTE, $H_{T}^{m}$, in grid cell *m* is shown in Equation (5):

(5) $$H_{T}^{m}=-\sum_{t=0}^{23}P_{t}^{m}\times \log P_{t}^{m}$$

Next, according to the area in which each grid cell is located, we calculate the TTE in each region. The calculation of the TTE, $H_{i}^{T}\left(1,2,\ldots ,16\right)$, in region *i* is shown in Equation (6):

(6) $$H_{i}^{T}=\frac{\sum_{m=1}^{M_{i}}H_{T}^{m}}{M_{i}}$$

### 4.3. Building the Coupling Coordination Degree Model

The degree of coupling refers to the degree of interaction between two or more systems. The degree of coupling coordination is based on the degree of coupling and further reflects the degree of coordinated development between systems [25]. This paper considers the PSE, TTE, and UDL as three systems. Based on the model of coupling coordination, we measure the coupling relationship and coupling coordination among these three systems. The specific measurement steps are as follows:

*Step 1*: Measure the UDL. As an objective evaluation method, the entropy method can not only avoid researchers’ subjective judgment, but also solve the problem of information overlap among multiple indicators. Therefore, according to the evaluation index system for UDL given in Table 1, we use the entropy method to determine the weight of each indicator. The calculation results are also shown in Table 1. The specific steps are as follows:
  *Step 1(a)*: Standardized processing. We standardize the original data to eliminate the influence of various index dimensions, sizes, and positive and negative directions. We use *X_ij_* and $X_{ij}^{\prime }$ to represent the value of indicator *j* and the standardized index value in region *i*, respectively.
  *Step 1(b)*: Calculate the regional UDL. First, calculate the information entropy *e_j_* of indicator *j*, as shown in Equation (7):
    (7) $$e_{j}=-\frac{1}{\ln16}\sum_{i=1}^{16}P_{ij}\times \ln P_{ij}\left(0\leq e\leq 1\right)$$

Here, *P_ij_* indicates the weight of index *j* in region *i*. The calculation formula is  Pij= Xij′∑i=116Xij″. Then, the UDL *U_i_* in region *i* is calculated as shown in Equation (8):

(8) $$U_{i}=\sum_{j=1}^{24}w_{j}\times X_{ij}^{\prime }$$

Here, *w_j_* is the weight of index *j*, and the calculation formula is wj=(1−ej)∑j=124(1−ej).

*Step 2*: Calculate the coupling degree $C_{i}$ among UDL, PSE and TTE in region *i*, as shown in Equation (9):
  (9) Ci={Ui×HiS×HiT[(Ui+HiS+HiT)/3]3}13

In order to further explore the pairwise coupling relationships among UDL, PSE and TTE, the calculation is as shown in Equation (10):

(10) CiUS={Ui×HiS[(Ui+HiS)/2]2}12, CiUT={Ui×HiT[(Ui+HiT)/2]2}12, CiST={HiS×HiT[(HiS+HiT)/2]2}12

In order to determine the coupling degree of the three systems (*C_i_*) or two systems ($C_{i}^{US},C_{i}^{UT},C_{i}^{ST})$, the coupling degree is divided into four levels based on previous research results: ① When $0\leq C_{i}<0.4$, there is low-level coupling, and the relationship between the three is extremely unstable. When $C_{i}=0$, there is no coupling, indicating that the three subsystems are unrelated. ② When $0.4\leq C_{i}<0.6$, the coupling is medium, and the relationship among the three systems is unstable. ③ When $0.6\leq C_{i}<0.8$, the coupling is at a relatively high level and the relationship among the three systems is basically stable. ④ When $0.8\leq C_{i}<1$, the coupling is at the highest level, and the relationship among them is very stable [39].

*Step 3*: Calculate the degree of coupling coordination *D_i_* among UDL, PSE and TTE in region *i*, as shown in Equation (11):
  (11) $$D_{i}=\sqrt{C_{i}\times T_{i}},\;T_{i}=\alpha U_{i}+\beta H_{i}^{S}+\varphi H_{i}^{T}$$
  where *T_i_* is the comprehensive evaluation index of the three systems in region *i*, and *α,β,φ* are undetermined coefficients that satisfy *α* + *β* + *φ* = 1. With reference to related studies [37,39,40], the coefficients are determined to be *α* = 0.2, *β* = 0.4 and *φ* = 0.4.

The calculation formula used to further explore the pairwise coupling coordination relationships among UDL, PSE and TTE is shown in Equation (12):

(12) $$D_{i}^{US}=\sqrt{C_{i}^{US}\times T_{i}^{US}},D_{i}^{UT}=\sqrt{C_{i}^{UT}\times T_{i}^{UT}},\;D_{i}^{ST}=\sqrt{C_{i}^{ST}\times T_{i}^{ST}}$$

Here, $D_{i}^{US}$ is the degree of coupling coordination between UDL and PSE at this time, $T_{i}^{US}=\alpha U_{i}+\beta H_{i}^{S}$, and the coefficients are determined to be *α* = 0.4, *β* = 0.6; $D_{i}^{UT}$ is the degree of coupling coordination between UDL and TTE at this time, $T_{i}^{UT}=\alpha U_{i}+\varphi H_{i}^{T}$, and the coefficients are determined to be *α* = 0.4, *φ* = 0.6; and $D_{i}^{ST}$ is the degree of coupling coordination between PSE and TTE at this time, $T_{i}^{ST}=\beta H_{i}^{S}+\varphi H_{i}^{T}$, and the coefficients are determined to be *β* = 0.5, *φ* = 0.5 [39,41].

The classification is based on the degree of coupling coordination, and the specific classification criteria are shown in Table 3 [42].

### 4.4. Building the Spatial Autocorrelation Model

#### 4.4.1. Global Spatial Autocorrelation Model

*Moran’s I* index is used to calculate the degree of global autocorrelation [43], and to analyze the spatial correlation and agglomeration of the UFMD and UDL. The calculation formula for *Moran’s I* index is as follows:

(13) $$I=\frac{n\sum_{i=1}^{n}\sum_{i^{\prime }\neq i}^{n}w_{{ii}^{\prime }}(D_{i}-\bar{D})(D_{i^{\prime }}-\bar{D})}{\sum_{i=1}^{n}\sum_{i^{\prime }\neq i}^{n}w_{ii^{\prime }}\sum_{i=1}^{n}{\left(D_{i}-\bar{D}\right)}^{2}}$$

where *n* is the number of regional units in the area, that is the total number of administrative districts (*n* = 16); $D_{i},D_{i^{\prime }}$ are the coupling coordination degree of regions *i* and *i^’^* (*i* = 1,2,…,16, *i ≠ i^’^*), respectively; $\bar{D}$ is the mean value of the coupling coordination degree; and $w_{ii^{\prime }}$ is the distance weight between regions *i* and *i^’^*. If 0 < *I* ≤ 1, it will indicate that the regions’ degrees of coupling coordination are positively correlated; if −1 ≤ *I* < 0, it will indicate a negative correlation; if *I* = 0, it will indicate there is no spatial correlation.

For *Moran’s I* index, the standardized *Z* statistic is generally used to test the significance of the spatial autocorrelation. It is calculated as follows:

(14) $$Z=\frac{I-E\left(I\right)}{\sqrt{VAR\left(I\right)}}$$

where *E*(*I*) and *VAR*(*I*) are the theoretical expectation and theoretical variance of *I*, respectively.

If *Z* is positive and significant, it will indicate that there is a significantly positive correlation, that is, that similar observations tend to gather together spatially. If *Z* is negative and significant, it will indicate that there is a significant negative correlation, that is, that similar observations tend to be dispersed. If *Z* is 0, the observed values will be distributed independently and randomly.

#### 4.4.2. Local Spatial Autocorrelation Model

Using the Getis-Ord Gi* index, the distribution of the high and low values of the coupling coordination degree can be identified in the process of evolution. The calculation formula is as follows:

(15) $$G_{i}^{*}=\frac{\sum_{i^{\prime }=1}^{n}w_{ii^{\prime }}D_{i^{\prime }}}{\sum_{i=1}^{n}D_{i}}$$

At the same time, there are corresponding standardized statistics, *Z*(*i*)^*^, for the index $G_{i}^{*}$:

(16) $$Z{\left(i\right)}^{*}=\frac{G_{i}^{*}-E\left[G_{i}^{*}\right]}{\sqrt{VAR\left[G_{i}^{*}\right]}}$$

where $E\left[G_{i}^{*}\right]$ and $\sqrt{VAR\left[G_{i}^{*}\right]}$ are the expectation and variance of $G_{i}^{*}$, respectively. If the value of *Z*(*i*)^*^ is positive and significant, it will indicate that the region around *i* is a cluster of high-value space. If the value of *Z*(*i*)^*^ is negative and significant, it will indicate that the region around *i* is a low-value spatial agglomeration.

## 5. Results and Discussion

### 5.1. Overview

**Figure 1:**
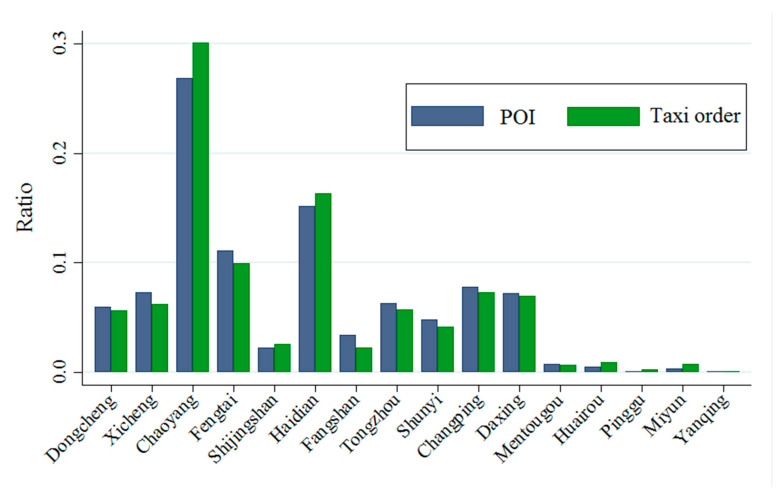
Distribution of POIs and taxi orders in different regions.

In a given area, the more POIs there are in both number and type, the higher the UFMD is [4]. Figure 1 shows the distribution of POIs and taxi orders in the 16 administrative districts of Beijing. On the whole, the distribution of POIs and taxi orders is imbalanced across the different regions, and the more POIs there are in each region, the more taxi orders, and vice versa. For example, POIs and taxi orders in Chaoyang, Haidian, and Fengtai are at high levels, while those in Pinggu, Miyun, and Yanqing are at lower levels.

It can also be seen from Figure 1 that the POIs and taxi orders are mainly distributed in the downtown areas (Dongcheng, Xicheng, Chaoyang, Fengtai, Shijingshan, Haidian), accounting for 68.60% and 70.74%, respectively. The main reasons for this are as follows: Firstly, the UFMD in the central urban area is high (according to data from the Beijing Municipal Bureau of Commerce, seven basic business functions offering convenience to people have been fully fulfilled in the six districts of the central city, putting their POIs at a high level), the infrastructure in these areas is relatively mature, and residences, offices, and leisure and entertainment facilities are relatively concentrated. Secondly, the economy of the central urban area is relatively developed (accounting for 69.4% of Beijing’s entire economy) and the population density is large (the proportion of Beijing’s entire population living in these areas is as high as 60%), which makes residents’ demand for taxis (thus the amount of taxi orders) high.

However, the numbers of POIs and taxi orders in the suburbs of Mentougou, Huairou, Pinggu, Miyun, and Yanqing are relatively small. The main reasons for this include the following: Firstly, most of the suburban areas, such as the Yanqing District, are located in mountain areas, where there are more green ecological areas, fewer commercial and other functional areas, which are quite dispersed, and a low degree of urban function mixing. Secondly, the economies of these areas are relatively backward, the industrial structure is imperfect, and the population density is low. The demand for taxis (number of taxi orders) is low.

**Figure 2:**
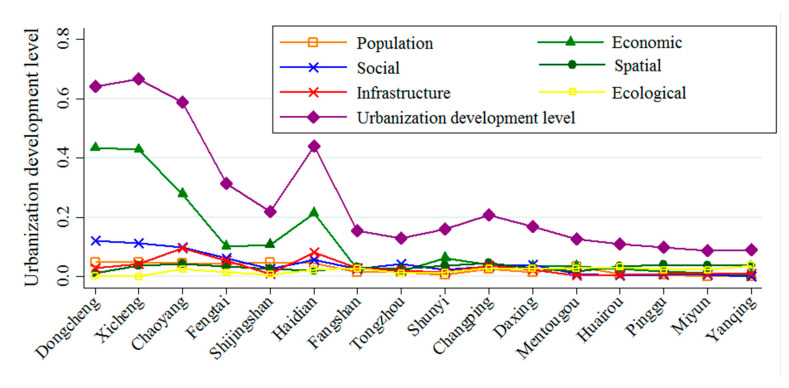
Distribution of urbanization development level across different regions.

We use Equation (8) to calculate the values of six urbanization indicators and the UDL in each region of Beijing. The results are shown in Figure 2. On the whole, the UDL and the six urbanization indicators, especially population urbanization, economic urbanization, and social urbanization, show the same trend from region to region, declining as we move from the central urban area to the outer suburbs. Among the six indicators, economic urbanization and spatial urbanization are the main factors affecting the UDL. The impact of population urbanization and infrastructure urbanization on the regional UDL is relatively small.

Looking at the UDLs in each region, the central urban areas (Dongcheng, Xicheng, Chaoyang, Fengtai, Shijingshan, Haidian) have the highest UDLs, which is because the population, economic, social, and infrastructure urbanization in these regions are all at a relatively high level, at about 3.08 times the level in other regions. However, in the suburbs of Pinggu, Yanqing and Miyun, the UDLs are lowest. Among the indicators that affect the UDL, only spatial and ecological urbanization are relatively high in the suburban areas, while the economic development level in these areas is low, with the economy in the suburban areas contributing less than 30.6% of Beijing’s total economy.

From the six indicators that affect the UDL, population, economic, and social urbanization are highest in Dongcheng, followed by Xicheng, and lowest in Yanqing. For example, in terms of economic urbanization indicators, Xicheng’s GDP and per capita disposable income are respectively 7.63 times and 2.46 times higher than those of Yanqing. Infrastructure urbanization is highest in Chaoyang and lowest in Mentougou, because the various infrastructures in Chaoyang are relatively sound. For example, there are 13.79 times more registered parking lots in Chaoyang than Yanqing. Spatial urbanization is highest in Changping and lowest in Dongcheng. Ecological urbanization is highest in Yanqing and lowest in Xicheng. This is mainly due to the fact that, in terms of spatial urbanization, the average commuting distance in the suburban areas is lower than that in the central urban areas, and the proportion of completed housing in construction areas is higher than that in the central urban area. In terms of ecological urbanization, the percentages of tree forest greening and waste innocuous treatment plants in suburban areas such as Yanqing are about 3.1 times those in the central urban areas.

### 5.2. Analyzing the Spatiotemporal Distribution of POIs’ Spatial Entropy and Taxis’ Temporal Entropy

**Figure 3:**
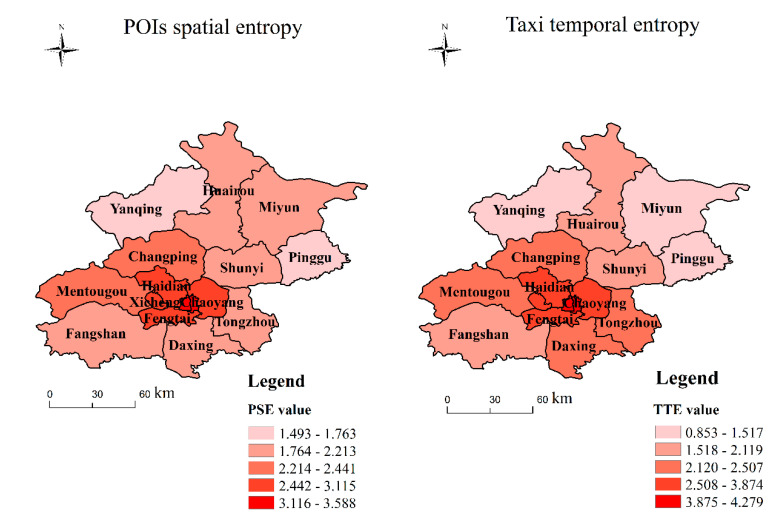
Distribution of POIs’ spatial entropy and taxis’ temporal entropy.

A higher PSE indicates more complexity in the types of functions in an area, while a higher UFMD indicates sounder development [4]. The higher is the regional UFMD, the higher is the residents’ demand for taxis, meaning that the TTE can also reflect the UFMD to a certain extent [4]. Using Equations (3) and (6) and ArcGIS, the PSE and TTE in each region of Beijing are calculated and visualized, as shown in Figure 3. On the whole, the PSE and TTE show significant regional imbalances, both appearing to decrease as we move from the central urban areas to the outer suburbs. To some extent, this indicates that the UFMD in the central urban areas is higher, while that in the outer suburbs is lower.

From the perspective of the individual regions, the PSEs and TTEs of the central urban areas (Dongcheng, Xicheng, Chaoyang, Haidian, Fengtai, and Shijingshan) are high (all above 2.87), while the entropy values in Yanqing and Pinggu are the lowest (about 1.35). There are two reasons for this: first, there are developed economies, various sound functional areas, balanced land use, and many different types of functional areas per unit area in the central urban areas. Second, the central urban area has a relatively large population density and relatively large passenger flow. There are large numbers of functional buildings such as commercial areas, residential areas, tourist attractions and universities. Residents have a greater demand for taxis at different times, which makes the TTE in the central urban areas high. In the outer suburbs, the infrastructure is relatively lagging behind, and the distribution of important areas such as high-tech industries and shopping mall buildings is relatively scattered. In addition, the population density in the outer suburbs is relatively low, there is roughly only one type of functional area per unit area, and the UFMD is low. For example, the numbers of hotels, bus stations, and subways per square kilometer in the central urban areas are respectively 1.99 times, 1.2 times, and 29.9 times more than those in the suburban areas. In addition, residents’ demand for taxis in the suburban areas is relatively low, making the TTE low.

**Figure 4:**
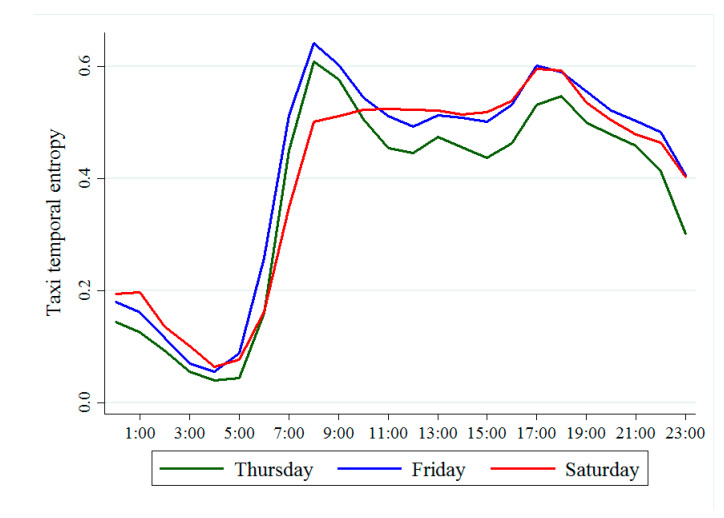
Distribution of taxi temporal entropy over time.

Meanwhile, Figure 4 shows the distribution of the TTE across time. On the whole, the TTE shows the same trend during both Thursday and Friday. People’s demand for taxis is higher in the morning and evening rush hours, with two travel peaks. However, there is no obvious morning peak on Saturday. This is because during the workdays, residents mostly travel to work, and the travel routes are relatively complicated. There are many types of functional areas represented by pick-up points, and residents have a higher demand for taxis. On weekends, residents are mainly moving around for leisure and entertainment purposes, and the travel routes are relatively simple. From 23:00 to 6:00 of the next day, when people are sleeping, travel is relatively infrequent, the demand for taxis relatively low, and the TTE also small.

### 5.3. Analyzing the Coupling Coordination Relationships among the UDL, PSE and TTE

#### 5.3.1. Coupling Degree Analysis in Different Regions

Now, using Equations (9) and (10), the coupling relationships among the UDL, PSE and TTE in each region of Beijing are measured. The results are shown in Table 4. The relationship among UDL, PSE, and TTE is referred to as UDL-PSE-TTE, that between UDL and PSE as UDL-PSE, and so on.

From Table 4, we can see that, except in Yanqing, the coupling relationships UDL-PSE-TTE, UDL-PSE and UDL-TTE are of the highest level in each region, and PSE-TTE is at a relatively high level, which indicates that the UFMD and UDL are in a stable state in all regions except Yanqing. However, all four coupling relationships in Yanqing are 0. According to the 2017 Beijing Statistical Yearbook data, all urbanization development indicators in Yanqing are at the lowest level, which indicates that the coupling relationships among UDL, PSE and TTE in Yanqing are at a low level. The relationship among the three systems is extremely unstable.

#### 5.3.2. Analyses of Coupling Coordination Relationships in Different Regions

**Figure 5:**
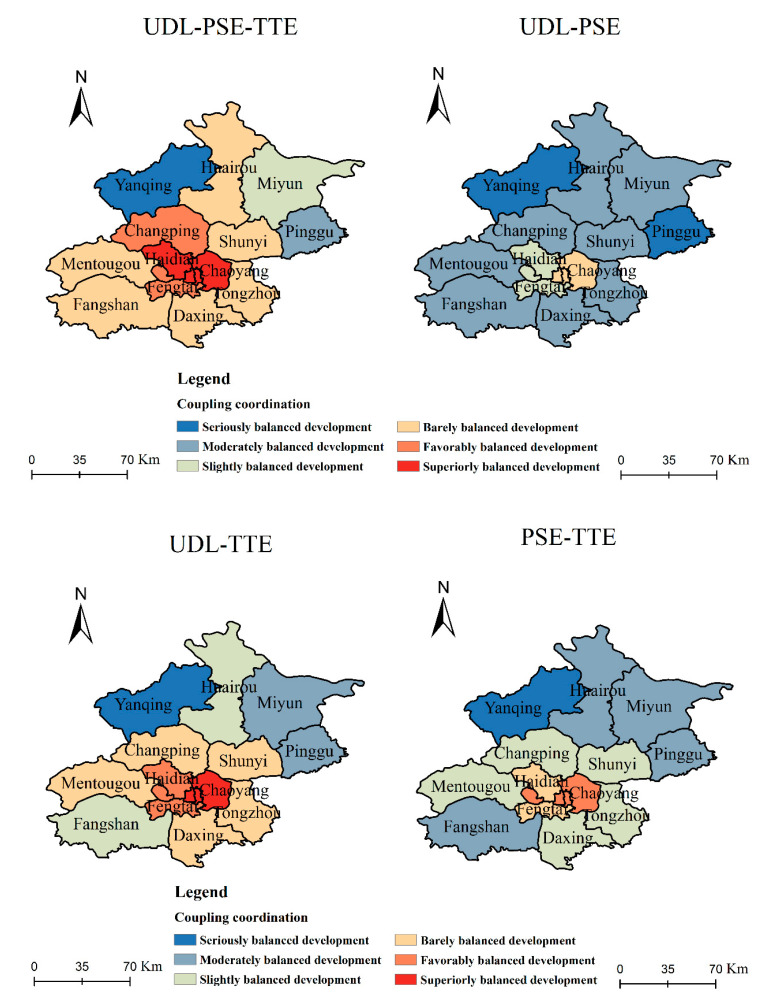
Spatial distributions of coupling coordination relationship across different regions.

According to Formulas (11) and (12), we calculate the coupling coordination relationships among UDL, PSE and TTE in each region of Beijing, as shown in Figure 5. From Figure 5, we can see that the relationship among UDL, PSE and TTE shows an imbalanced distribution across the various regions.

On the whole, the three types of coupling coordination in the central urban areas (Dongcheng, Xicheng, Chaoyang, Haidian, Fengtai) are in balanced development, while in the outer suburbs (Yanqing, Miyun, Pinggu) they are in unbalanced development. The reasons are as follows: First, the UDL indicators in the central urban areas are significantly higher than those in the outer suburbs. For example, the number of corporate units, per capita consumption expenditure, and number of beds in health institutions in the central urban areas are respectively 24.91 times, 1.09 times, and 1.86 times the figures in the outer suburbs. Second, the urbanization facilities in the central urban areas are better. For example, the number of recorded parking lots, bus stops, and commodity trading markets in the central urban areas are respectively 3.48 times, 1.20 times, and 1.16 times those in the outer suburbs. Third, residents have greater demand for taxis in the central urban areas. As can be seen from Figure 1, taxi orders in the central urban areas are 2.42 times those in the outer suburbs.

Next, we analyze the overall and pairwise coupling coordination relationships among UDL, PSE and TTE in various regions. The overall coupling coordination relationship among the three systems falls into the range of balanced or transitional development in up to 87.5% of regions, which indicates that the UDL and UFMD in most regions of Beijing are in coordinated development. In detail, in the central urban areas the relationship is in balanced development; in suburban areas such as Fangshan and Daxing it is in transitional development; and only in Yanqing and Pinggu is it in unbalanced development. There are two reasons for this. First, the UFMD of the central urban areas is significantly higher than that of suburban areas. For example, the PSE and TTE of the central urban areas are 0.92 times and 1.19 times higher than those of the suburban areas. Second, the indicators of the UDL in the balanced developmentare much higher than those in the suburban areas. From the perspective of the UDL in each region, that in the central region is 2.15 times higher than that in the suburban areas.

Looking at the pairwise coupling coordination relationship, the distribution of the UDL-PSE, UDL-TTE, and PSE-TTE coupling coordination relationships is obviously different in each region (The coupling coordination degree results are from Figure 5). The coupling coordination relationship of UDL-PSE is more often falling into the unbalanced development range, up to 62.5% of the time, which indicates that the UDL and PSE are not coordinated in most areas of Beijing. In terms of UDL-TTE, the areas in balanced or transitional development account for 81.25% of the total area, while only Yanqing and Pinggu are in the unbalanced development category. With regards to PSE-TTE, most areas are in the transitional development category, accounting for 43.75% of the total area. Only in Dongcheng, Xicheng, Chaoyang and Shijingshan is the PSE-TTE coupling coordination relationship in the balanced development range, and in Huairou, Pinggu, Miyun and Yanqing it is in the unbalanced development range. From the distributions of UDL-PSE, UDL-TTE, and PSE-TTE, we can deduce the following: firstly, the UDL and PSE in most regions are in unbalanced development; secondly, the UDL and TTE, and PSE and TTE are in the transitional development stage in most areas.

### 5.4. Analyzing Spatial Correlation Characteristics of Coupling Coordination Relationship between UFMD and UDL

In order to study the spatial agglomeration pattern and the evolution of the coupling coordination between the UFMD and UDL in the various regions of Beijing, we use the global autocorrelation method to calculate the global autocorrelation index (*Moran’s I*), as shown in Table 5.

According to Table 5, the global autocorrelation index of the coupling coordination relationships among PSE, TTE, and UDL (and the pairwise relationships) is always above 0.7, and the *Z*(*I*) value is relatively high, which indicates that the coupling coordination between the UFMD and UDL has a strong positive correlation in space, and the spatial agglomeration situation is more obvious.

**Figure 6:**
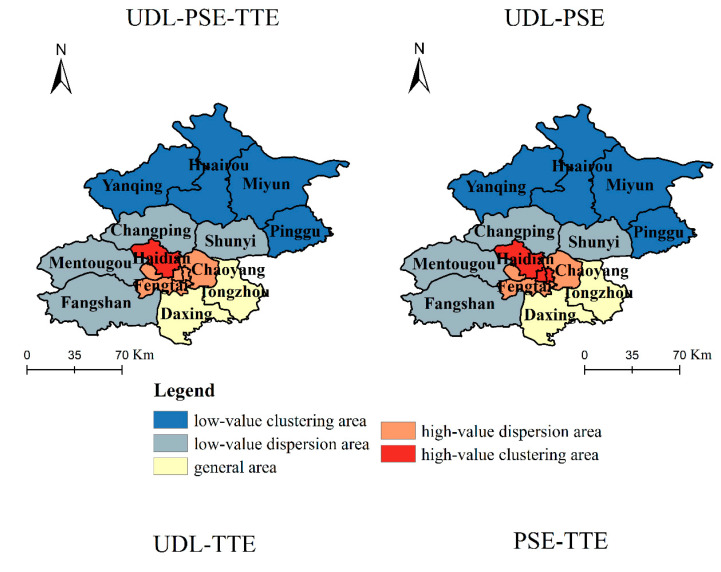

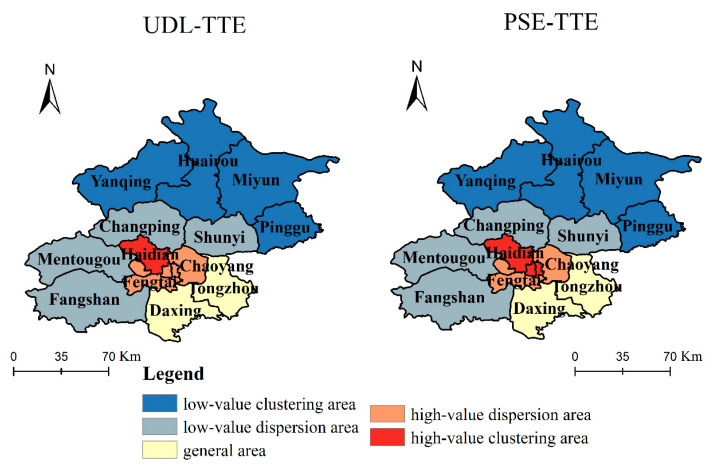
Spatial distribution of high-low-value clustering points with different degrees of coupling coordination.

To further explore the coupling coordination agglomeration phenomenon and identify the high- and low-value clustering distribution of PSE, TTE and UDL, the local spatial autocorrelation method is now used to calculate the index Getis-Ord Gi* in each region. Then the regions are divided into five levels: low-value clustering, low-value dispersion, general, high-value dispersion and high-value clustering, according to the natural breaking point method. Then, based on the clustering results for the coupling coordination relationship between the UFMD and UDL, spatial distribution maps of the clustering points with high and low coupling coordination relationships in each region of Beijing are drawn, as shown in Figure 6.

On the whole, the spatial distribution of the high- and low-value clustering points of the overall coupling coordination relationship is similar to that of the pairwise coupling coordination relationships. Specifically, the numbers of low-value clustering, low-value dispersion, general, high-value dispersion and high-value clustering areas for UDL-PSE-TTE and UDL-PSE are 4, 4, 2, 5 and 1, while the numbers for UDL-PSE and USE-TTE are 4, 4, 2, 4, and 2. These results show that the coupling coordination relationship between the UFMD and UDL has obvious regional imbalances.

Looking at the various regions, Haidian, Dongcheng, Xicheng, Chaoyang, Fengtai, and Shijingshan are high-value clustering areas and high-value dispersion areas, which are the areas with a high level of coupling coordination between UFMD and UDL. Daxing and Tongzhou are general areas of coupling coordination; Shunyi, Changping, Mentougou, and Fangshan are low-level dispersion areas of coupling coordination; and Pinggu, Miyun, Huairou, and Yanqing are low-value clustering areas of coupling coordination. This regional imbalance results from the following: First, the UFMD in central urban areas such as Haidian and Chaoyang is relatively high. For example, Haidian has many colleges and universities, Chaoyang has a large number of CBDs, and Dongcheng and Xicheng have a lot of historic sites and tourist attractions. Second, the UDL in the central urban areas is relatively high. For example, the gross regional product and per capita disposable income in the central urban areas are approximately 1.62 times and 1.12 times higher than those in the outer suburbs. Third, there are large numbers of permanent residents and foreign tourists in the central urban areas, which leads to a large passenger flow, and significantly higher demand for taxis from residents than in the suburban areas. For example, the permanent resident population of the central urban areas is 1.48 times that in the outer suburban areas, and the number of inbound tourists is 9.3 times the number in the outer suburbs.

## 6. Discussion

Compared with similar previous studies, our innovation is mainly reflected in the following three aspects. First, the calculation of the degree of urban function mixing. Compared with the previous studies on the measurement of the urban function mixing degree, our study uses multi-source data and designs a more comprehensive index system which is helpful for analyzing and evaluating the urban function mixing degree from various perspectives. For example, in terms of data, previous studies have mainly used POI data to classify and evaluate urban functional areas [13]. However, we not only use urban POI data, but also taxis’ GPS trajectory data, resulting in three outcomes. Firstly, from the categorization of physical space POI data into different functional areas, we can preliminarily judge the degree of mixing of different functional spaces in the city. Secondly, we use the taxis’ GPS data in different time periods to analyze the actual utilization of various functional areas by actors in the city. Finally, the combination of two outcomes make the measurement results more accurate and reliable. In terms of measurement methods, most previous studies have measured the mixing of urban functional areas by calculating the POI density [14] or POI spatial entropy [19]. We, however, measure two kinds of data, including urban POIs’ spatial entropy and taxis’ temporal entropy in a specific area. This is because the urban function mixing degree in a city is difficult to fully analyze with only one kind of data. Specifically, the urban function mixing degree is not only related to the POI, representing the type of urban land use, but also to the city’s traffic behavior. Thus, by introducing taxis’ GPS trajectory data, we can describe the function mixing degree in specific areas in more detail.

Second, the differences in research area and methods. Most of the previous studies have looked at the causes of unbalanced urbanization development in different cities from the perspective of the whole city; while most no research has analyzed the factors affecting this uneven development from an inter-regional perspective. The results show that, in different areas of the city, the urbanization development level and function mixing degree are obviously unbalanced. The three types of coupling coordination degrees in the central urban area are all in balanced development, while in the outer suburbs they are all in unbalanced development. The higher the degree of spatial agglomeration of coupling coordination, the higher the urban function mixing degree and urbanization development level. Moreover, the higher the coupling and coordination among the three and pairwise, and vice versa. In the past, the coupling coordination degree model was mainly applied in the fields of economy, environment, etc. Nevertheless, regarding the coordination relationship between the urban spatial structure and the level of urbanization development, the coupling coordination degree model has not yet been studied. We use the coupling coordination degree model to measure the coordination relationship between the urbanization development level and the urban function mixing degree. In addition, in order to more clearly reveal the spatial distribution pattern of the coupling coordination among different areas of the city, we use the spatial autocorrelation analysis method to analyze the spatial correlation characteristics of the coupling coordination, and further understand the unbalanced development of urban areas.

Third, the internal reasons for the unbalanced development of urban regions. The incoordination between urbanization development level and urban spatial structure has attracted public attention, which makes its internal factors especially worth studying. Therefore, the departments concerned could better take measures from a macro and micro perspective to solve the problem of uncoordinated urban development. On the one hand, the urbanization indicators of different areas of the city are in an unbalanced state. For example, the economic urbanization, population urbanization, and social urbanization in the central areas of the city are all at a relatively high level, while only the ecological urbanization of the outer suburbs is at a relatively high level. On the other hand, the urban land utilization rate shows obvious regional discrepancies. This is mainly reflected in the sound infrastructure and high land resource utilization efficiency in the central urban areas, while the outer urban area is large, lacks various types of public transportation and other infrastructure, and has fewer and scattered functional areas. Therefore, it is necessary to rationally use land resources in central urban areas to improve the efficiency of infrastructure utilization, while in the outer suburbs of cities, regional construction should be strengthened to further meet the living needs of urban residents, by for example activating communities, improving the accessibility of services and facilities, reducing the use of private cars, increasing residents’ sports activities, etc.

## 7. Conclusions

The problem of uncoordinated development between urban scale expansion and the construction of functional areas is common to various cities in China. In the process of urbanization, coordinated development between urban spatial expansion and land use balance is a very important part of promoting the “healthy” development of cities. This requires cities to improve their urban land use efficiency and achieve rational planning of the urban spatial structure. Thus, a basic element of policy reform is to accurately measure the coupling coordination between the function mixing degree of different urban areas and the urbanization development level. Taking Beijing as an example, based on urban POI data and taxis’ GPS trajectory data, we use the urban POIs’ spatial entropy and taxis’ temporal entropy to measure the urban function mixing degree. Finally, we build a coupling coordination degree model to measure and evaluate the coupling coordination degree between the latter and the urbanization development level. Our research provides a method for quantitatively evaluating the urban function mixing degree and proposes a generalized model for evaluating the coupling coordination relationship between it and the urbanization development level. The quantitative methods and models used in the research can be generalized to other cities, which will help different cities to formulate corresponding management policies according to local conditions.

The study finds that the higher is the spatial agglomeration degree, the higher are the urban function mixing degree and urbanization development level, and the higher the coupling coordination level among all three and any two of them, and vice versa. In the process of urbanization, the overall land utilization rate of Chinese cities is still at a low level, especially in the suburbs, where the urban function mixing degree and urbanization development level are low and the development is imbalanced. This shows that Chinese cities should improve the efficiency of land resource utilization, focus on improving the construction of functional areas in outer suburbs, and speed up the coordinated development of urbanization. Urban development is a process of urban expansion and reconstruction. Improving the efficiency of urban land use and promoting the coordinated and healthy development of cities has become a topic of increasing concern.

Coordinated development between the urban function mixing degree and urbanization development level is a dynamic process. Analyzing the dynamics of this in different regions is not only conducive to the improvement of urban functional buildings, but also to the sustainable development of urbanization. Relevant departments should formulate relevant urban planning policies according to the spatial agglomeration degree and the coupling coordination between the functional mixing degree and the urbanization development level, so as to improve the functional construction in each region of the city. The urbanization development process in different regions of the city should be adjusted according to the actual situation and differentiated development strategies should be implemented for different regions. For example, the level of modernization should be improved for urban areas, and the level of urbanization should be improved for the outer suburbs of cities. In this way, the urban areas and outer suburbs of a city will gain complementary advantages, mutual promotion, and coordinated development, promoting the healthy, coordinated and sustainable development of the entire city.

Our research currently has certain limitations. Firstly, the selection of evaluation indicators for the urbanization development level mainly relies on the indicator system found in the past literature and the existing Chinese statistical data. Therefore, our selection is not comprehensive and specific, and we need more open source data to support the selection of other indicators, such as the mileage of urban transportation lines and commuting times. Secondly, due to availability restrictions, the multi-source data used in this article is not yet complete, and some social factors, such as cultural customs, architectural features, and local policies, are difficult to quantify. Since a city is a complex system, a city’s data is formed from the interaction of multiple dimensions of complex data and has the characteristics of multiple heterogeneity. In future research, we can use multi-source data to supplement the research on the urbanization development level and urban function mixing degree, and analyze the factors affecting urbanization development from multiple dimensions. In addition, the impact of different urban transportation systems on the urbanization development level would be worthy of further exploration.

## Figures and Tables

**Table 1:** Comparison of synchronous research on urban function mixing egree.

Classification Criterion			Study and Topic	
***Data***	Vehicle trajectory data		Gao, Q. (2019) [11]	Classify urban functional areas
			Wu, Q. (2018) [12]	
	Vehicle trajectory data and POIs		Kang, Y. (2018) [13]	Determine the spatial distribution and combination of urban functional areas
	Sina Weibo’s POIs and check-in data		Long, Y. (2013) [14]	Evaluate the function mixing degree
				Measure the function mixing degree in a specific area
***Method***	Objective weight	Entropy weight and mean square deviation method	Zhao, M. (2018) [15]	Measure the functional intensity of urban development land
			Yuan, J. (2012) [16]	Study the urban function mixing degree in different areas
		Land use mixing index	Frank, L.D. (2004) [17]	Calculate the urban function mixing degree in each grid
		Probabilistic model	Liu, X. (2017) [18]	Infer buildings’ mixed functions
		Spatial entropy	Decraene, J. (2013) [19]	Measure the degree of diffusion in functional areas
		The difference index	Decraene, J. (2013) [19]	Quantify the mixing degree of different functional areas in the space
	The combination of subjective weight with objective weight	The measurement method for the life/work/access triangle	Dovey, K. (2017) [20]	Reveal functional combination changes at different scales by means of map technology
				Explain and measure the functional mixing degrees in different cities

**Table 2:** Evaluation index system of urbanization development level.

Target Layer	Criteria Layer	Index Layer	Unit	Weight
Urbanization development level	Population urbanization	Percentage of urban population	%	0.025
		Percentage of nonagricultural population	%	0.022
	Economic urbanization	GDP	10 thousand yuan /person	0.048
		Private cars	number/person	0.059
		Per capita disposable income	yuan	0.039
		Per capita consumption expenditure	yuan	0.037
		Total wages of employees	10 thousand yuan /person	0.052
		Number of corporate units	number/km²	0.088
		Number of employees at the end of the year	person/km²	0.092
		Number of inbound tourists	10 thousand people	0.096
		Number of starred hotels	number	0.039
	Social urbanization	Number of beds in health institutions	number	0.032
		Total social investment in fixed assets	100 million yuan	0.033
		Number of bus stops	number/km²	0.023
		Number of subways	number/km²	0.090
	Spatial urbanization	Commuting distance	km	0.011
		Distance from city center	km	0.025
		Percentage of completed housing in the construction area	%	0.013
		Number of commodity trading markets	number	0.030
	Infrastructure urbanization	Electricity consumption	million kilowatt hours	0.033
		Number of recorded parking lots	number	0.047
		Number of adoptive units	number	0.027
	Ecological urbanization	Percentage of tree forest greening	%	0.017
		Number of waste innocuous treatment plants	number	0.022

**Table 3:** Classification standard for degree of coupling coordination.

Class	D	Subclass
Balanced development (Acceptable interval)	0.8 < *D* ≤ 1.0	Superiorly balanced development
	0.6 < *D* ≤ 0.8	Favorably balanced development
Transitional development (Transitional interval)	0.5 < *D* ≤ 0.6	Barely balanced development
	0.4 < *D* ≤ 0.5	Slightly unbalanced development
Unbalanced development (Unacceptable interval)	0.2 < *D* ≤ 0.4	Moderately unbalanced development
	0.0 ≤ *D* ≤ 0.2	Seriously unbalanced development

**Table 4:** Coupling relationships in different regions of Beijing.

Region	UDL-PSE-TTE	UDL-PSE	UDL-TTE	PSE-TTE
Dongcheng	0.9795	0.8763	0.9759	0.7738
Xicheng	0.9832	0.8695	0.9805	0.7770
Chaoyang	0.9863	0.8533	0.9799	0.7573
Fengtai	0.9359	0.9477	0.9123	0.7647
Shijingshan	0.8576	0.9920	0.8163	0.7546
Haidian	0.9717	0.8852	0.9628	0.7572
Fangshan	0.9522	0.8755	0.9449	0.7143
Tongzhou	0.8948	0.9775	0.8539	0.7520
Shunyi	0.9442	0.9307	0.9176	0.7456
Changping	0.9361	0.9285	0.9171	0.7417
Daxing	0.9269	0.9592	0.8945	0.7618
Mentougou	0.8661	0.9827	0.8189	0.7275
Huairou	0.8915	0.9845	0.8643	0.7820
Pinggu	0.9931	0.7416	0.9923	0.6794
Miyun	0.9049	0.9415	0.9255	0.7728
Yanqing	0.0000	0.0000	0.0000	0.0000

**Table 5:** Global autocorrelation of coupling coordination degree.

D	UDL-PSE-TTE	UDL-PSE	UDL-TTE	PSE-TTE
*Moran’s I*	0.745	0.784	0.759	0.705
*Z*(*I*)	5.291	5.504	5.361	5.029
*P*(*I*)	0.000	0.000	0.000	0.000

## Data Availability

The data used to support the findings of this study are available from the corresponding author upon request.
